# Failure of ceftolozane‐tazobactam salvage therapy in complicated pneumonia with lung abscess

**DOI:** 10.1002/ccr3.1612

**Published:** 2018-05-28

**Authors:** Paul O. Lewis, David B. Cluck, Jennifer L. Tharp, Matthew A. Krolikowski, Paras D. Patel

**Affiliations:** ^1^ Department of Pharmacy Johnson City Medical Center Johnson City TN USA; ^2^ Department of Pharmacy Practice Bill Gatton College of Pharmacy East Tennessee State University Johnson City TN USA; ^3^ Division of Infectious Diseases Quillen College of Medicine East Tennessee State University Johnson City TN USA

**Keywords:** ceftolozane‐tazobactam, pneumonia, *Pseudomonas aeruginosa*, resistance, treatment failure

## Abstract

Treatment of *Pseudomonas aeruginosa* remains challenging, despite the availability ceftolozane‐tazobactam. We report a treatment failure with ceftolozane‐tazobactam salvage therapy for pneumonia complicated by lung abscess. Drug resistance, dose selection, and source control are possible contributing factors. Ceftolozane‐tazobactam susceptibility testing should precede therapy and consideration should be given to dosing selection.

## 1 INTRODUCTION

Ceftolozane‐tazobactam is a new oxyimino‐cephalosporin/beta‐lactamase inhibitor combination currently approved by the United States (US) Food and Drug Administration (FDA) for the treatment of complicated urinary tract infections and complicated intraabdominal infections at a dose of 1.5 g intravenous (IV) every 8 hours.^1^ This agent has gained notable attention for enhanced activity against resistant Gram‐negative organisms, specifically *Pseudomonas aeruginosa*.^2^ *Pseudomonas aeruginosa* is problematic due to numerous resistance mechanisms against beta‐lactams, including loss of porin channels, efflux pumps, and beta‐lactamase production (see Table 1).^2^ Ceftolozane contains structural differences that allow it to remain active against these mechanisms and is currently the most active beta‐lactam directed against *P. aeruginosa*.^3^

**Table 1:** Notable resistance mechanisms produced by *Pseudomonas aeruginosa* 2, 11, 13, 16, 17, 18, 19

	Mero	Imi	Cfp	Azt	PT	CT
Beta‐lactamases						
Class A carbapenemase (KPC)	R	R	R	R	R	R
Class B (MBL, NDM, VIM, IMP)	R	R	R		R	R
Class C (AmpC)^a^	r	r	r	R	R	r
Class D (OXA‐type ESBL)			R	R	r	
Class D (OXA‐type carbapenemase)	R	R	R	R	R	R
Efflux pumps						
MexAB‐OprM	R		R	R	R	
MexCD‐OprJ	R		R		R	
MexEF‐OprN	R	R	R	R	R	
MexXY‐OprM	R		R	R	R	
Other mechanisms						
Loss of OprD (porins)	r	R				

Azt, aztreonam; Cfp, cefepime; CT, ceftolozane‐tazobactam; Imi, imipenem; Mer, meropenem; PT, piperacillin‐tazobactam; r, partial resistance; R, complete resistance.

a Includes *Pseudomonas*‐derived cephalosporinases (PDC) variants.

While not FDA approved, a phase I trial demonstrated ceftolozane‐tazobactam achieved higher epithelial lung concentrations than piperacillin‐tazobactam in 51 healthy adults.^4^ A phase III trial registered with the National Institute of Health (NCT018539) for the treatment of ventilator‐associated pneumonia was terminated early to redirect all efforts to a larger trial within the clinical development program (NCT02070757) and is currently enrolling patients with ventilated nosocomial pneumonia.^5^, ^6^ Notably, these trials are evaluating a higher dose of 3 g IV every 8 hours. Several case reports have also described successful use of ceftolozane‐tazobactam in pneumonia, secondary to multi‐drug resistant *P. aeruginosa* (MDRPA).^7^, ^8^, ^9^, ^10^

Failures have been observed with ceftolozane‐tazobactam when used off‐label. Caston and colleagues studied 6 patients with pneumonia, 2 resulting in deaths and one resulting in a clinical cure but microbiological persistence at 30 days.^8^ These patients were confounded by septic shock and multiple medical comorbidities, including one with a lung transplantation. Munita and colleagues studied 18 patients with pneumonia, 7 of whom were considered treatment failures.^9^ We report our experience and lessons learned with a treatment failure in a patient treated for pneumonia complicated by lung abscess.

## 2 CASE PRESENTATION

We present the case of a 53‐year‐old female who transferred from an outside facility requiring a higher level of care, due to worsening pneumonia with possible abscess and the need for cardiothoracic surgery (CTS) consultation. Past medical history included previous breast cancer post‐lumpectomy and radiotherapy, remote history of vulvar and rectal cancer post wide‐debulking, chronic obstructive pulmonary disease, and ventilator‐dependent respiratory failure with tracheostomy. Home medications included albuterol/ipratropium nebulizer, alprazolam, amlodipine, aripiprazole, budesonide/formoterol metered dose inhaler, citalopram, tamoxifen, tiotropium inhaler, trazodone, and oxycodone. She reported an allergy to nonsteroidal anti‐inflammatory drugs. The patient initially presented to another facility complaining of fever, diarrhea, shortness of breath, and increasing oxygen demands. On examination, the patient was not in acute distress. All systems were negative except for diminished lung sounds with rhonchi bilaterally. A chest X‐ray demonstrated left upper lobe pneumonia. Blood cultures were drawn, and a sample of tracheostomy secretions was sent for culture and sensitivities. She was initiated on vancomycin and piperacillin‐tazobactam for treatment of healthcare‐associated pneumonia. The patient reported a history of *Clostridium difficile* colitis and was started on oral vancomycin and IV metronidazole. On day 2, a bronchoscopy was performed with washings sent for culture. Both respiratory cultures grew MDRPA while blood cultures remained negative. The susceptibility profile is outlined in Table 2. Piperacillin‐tazobactam was switched to meropenem at 2 g IV every 8 hours, and inhaled tobramycin was added. A repeat bronchoscopy was performed on day 12 due to mucus plugging and lack of clinical response. This culture grew persistent MDRPA, necessitating the addition of IV tobramycin on day 16. Inhaled tobramycin was switched to inhaled colistin on day 20. On day 23, tobramycin IV and metronidazole were discontinued and the meropenem dose was decreased.

**Table 2:** Summary of the patient cultures and sensitivities^a^

Day	Day 1	Day 2		Day 12		Day 24		Day 32	Day 36
Site	Tracheostomy	Bronchoscopy		Bronchoscopy		Pleural Fluid		Pleural Fluid	Bronchoscopy
Organism	*Pseudomonas aeruginosa*	*Pseudomonas aeruginosa*		*Pseudomonas aeruginosa*		*Pseudomonas aeruginosa*		*Pseudomonas aeruginosa*	*Acinetobacter baumanii*
Isolate no.	1	1	2	1	2	1	2	1	1
Cefepime	16 I	16 I	≥64 R	8 S	≥64 R	≥64 R	16 I	≥64 R	≥64 R
Gentamicin	4 I	4 I	4 I	≤1 S	4 I	4 I	2 S	8 I	8 I
Levofloxacin	≥8 R	≥8 R	≥8 R	≥8 R	≥8 R	≥8 R	≥8 R	4 I	4 I
Imipenem								≥16 R	≥16 R
Meropenem	≥16 R	≥16 R	≥16 R	≥16 R	≥16 R	≥16 R	≥16 R	≥16 R	
Pip/tazo	32 S	32 S		32S					
Tobramycin	≤1 S	≤1 S	≤1 S	≤1 S	≤1 S	≤1 S	≤1 S	≤1 S	≤1 S
Amikacin								128 R	128 R
Cef/tazo								12 NS	
Colistin								0.094 S	0.094 S

Cef/tazo, ceftolozane‐tazobactam; I, intermediate; NS, nonsusceptible; Pip/tazo, piperacillin‐tazobactam; R, resistant; S, susceptible.

a All tested susceptibilities are provided. If not listed, susceptibilities were either not performed or not reported.

On day 24, a 12‐French chest tube was successfully placed into the left upper lobe abscess. Approximately 30 mL of purulent material was aspirated and sent for culture. Growth from this drainage continued to demonstrate MDRPA. One day after chest tube insertion, a computed tomography (CT) scan of the chest demonstrated significantly diminished fluid component of the left upper lobe abscess with drain positioned in the air‐containing component; residual left upper lobe and left lingular pneumonia and pulmonary emphysema was also noted. The patient failed to improve and on day 28 ceftolozane‐tazobactam was initiated at 1.5 g IV every 8 hours. A repeat chest CT scan on day 30 showed a decrease in the left upper gas‐fluid collection, but persistent dense pneumonia of the left upper lung and a new smaller infiltrate in the left lower lobe.

Two days after the repeat chest CT, the patient was transferred to our facility for worsening pneumonia, requiring a higher level of care and CTS consultation. Abnormal clinical examination findings included diffusely diminished lung sounds, rales in the left lower lobe, a left pigtail chest tube drain, tachycardia with heart rate of 120 beats per minute, and tachypnea with a respiratory rate of 22 breaths per minute. The patient's chest tube was intact with a small amount of thick, white drainage, which was sent for culture, along with 2 sets of blood cultures. Ceftolozane‐tazobactam 1.5 g IV every 8 hours was continued, and meropenem was stopped. The patient was initiated on tobramycin 280 mg (7 mg/kg) IV every 24 hours. CTS diagnosed the condition as a pulmonary abscess with bronchopulmonary fistula (BPF). The patient was deemed a poor surgical candidate, and continued medical management and drainage via chest tube was recommended. Repeat cultures from the pleural fluid drainage continued to grow MDRPA, while blood cultures remained negative. Susceptibility testing for ceftolozane‐tazobactam was requested and performed using an investigational elipsometer (E)‐strip. On day 36 overall (day 5 post‐transfer), a repeat bronchoscopy was performed. Culture of the washings grew *Acinetobacter baumaunii*. Additionally, the results from the ceftolozane‐tazobactam E‐strip for *Pseudomonas* finalized, demonstrating an MIC of 12 mg/L (nonsusceptible). Chest CT on day 37 demonstrated mild interval increase in the air‐fluid collection size, drain in place, persistent extensive left upper lobe pneumonia, and mild improvement in the lingula and left lung base. With no improvement and the growth of *Acinetobacter*, ceftolozane‐tazobactam was discontinued on day 39, after 12 days total. Meropenem at 1.5 g (pediatric dosing of 40 mg/kg) IV every 8 hours and colistin 100 mg (2.5 mg/kg) IV every 12 hours were started. After 14 days of meropenem and colistin without improvement, all therapy was stopped and the patient was transitioned to comfort care and ultimately expired.

## 3 DISCUSSION

This case describes treatment failure with ceftolozane‐tazobactam for pneumonia with pulmonary abscess. Several contributing factors warrant further discussion. In our case, the reported MIC to ceftolozane‐tazobactam was 12 mg/L. The prescribing information for ceftolozane‐tazobactam defines susceptible as ≤ 4 mg/L for *P. aeruginosa*.^1^ Susceptibilities prior to starting treatment were not available. This raises an unanswerable question whether resistance was present at baseline or had developed during therapy.

Fraile‐Ribot and colleagues reported the emergence of ceftolozane‐tazobactam resistance during the treatment of surgical site infection with ceftazidime.^11^ Polymerase chain reaction sequencing performed on the initial culture and the resistant culture demonstrated that resistance was due to an amino acid residue insertion at D149 on _*bla*_OXA‐2 beta‐lactamase, now designated OXA‐539.^11^ This report is concerning for multiple reasons. In vivo resistance to novel cephalosporins is possible even without previous exposure.^11^ An extended spectrum beta‐lactamase emerged from a narrow spectrum beta‐lactamase (OXA‐2).^11^ Furthermore, this gene is highly transmissible, associated with higher virulence factor (exoU cytotoxin), and has already been detected in 14 different countries.^12^ While our patient was exposed to multiple beta‐lactams prior to treatment with ceftolozane‐tazobactam, the patient had not been exposed to any cephalosporins. Haidar and colleagues have also described ceftolozane‐tazobactam failures in a single center retrospective analysis.^13^ Notably, 18 of 21 cases in their analysis were respiratory tract infections.^13^ The failure rate in the study approached 30% with the majority of these cases being attributed to pneumonia.^13^ Moreover, the authors describe emergence of resistance while on therapy, citing ampC overexpression and/or point mutations as possible mechanisms contributing to the aforementioned failures.^13^ This is the first study to utilize whole genome sequencing to characterize resistance.^13^

The current FDA labeling only includes 1.5 g IV every 8 hours, while the proposed dose for pneumonia is 3 g IV every 8 hours.^1^, ^6^ Haidar and colleagues observed that dose was not associated with clinical failure or 90‐day mortality.^13^ In fact, only the approved dose of 1.5 g was found to result in success in some cases. Munita and colleagues were also challenged by this debate as they reported variations in their dosing.^9^ Ultimately, our decision was made to continue 1.5 g every 8 hours, given the patient's weight of 40 kg and poor nutritional status. Protein binding is 16%‐21%, and volume of distribution is 13.5 L following a single dose of 1.5 g in healthy adults. The prescribing information does not include pharmacokinetic data in patients with low body weight.^1^

Source control may have contributed to treatment failure. It remains a critical step in managing a wide variety of infectious processes. The course was complicated by the presence of an abscess and BPF, making the decision for more aggressive surgical intervention difficult. Infectious Diseases Society of America community‐acquired pneumonia guidelines state a thoracentesis should be performed whenever possible in patients with empyema and pleural effusion as these often represent causes for nonresponse.^14^ The chest tube allowed for continuous drainage. Furthermore, once the underlying lung disease improves, BPFs usually resolve without surgery.^15^ Ultimately, risk‐benefit analysis did not favor a more invasive surgical intervention.

In conclusion, ceftolozane‐tazobactam, preceded by piperacillin‐tazobactam, meropenem, tobramycin, and colistin, for the treatment of pneumonia in our patient was not successful. The patient's history was complicated by multiple medical comorbidities. Resistance to this agent was reported during treatment. Despite the success reported in other cases, caution is still warranted when using this agent for off‐label indications, especially when source control cannot be obtained. Consideration must also be given to which dose to use, the lower FDA‐approved dose or the higher dose, currently in clinical trials. Furthermore, *P. aeruginosa* should have susceptibilities performed to ceftolozane‐tazobactam, when available, prior to initiating therapy and upon re‐isolation.

## AUTHORSHIP

POL: wrote the introduction and case description. DBC, MAK, and JLT: wrote the discussion and added details of the case. PDP: was involved with the clinical care of the patient and edited the manuscript.

## CONFLICT OF INTEREST

No authors have any conflicts of interest to report. All authors contributed substantially to the concepts, writing, editing, and approval of this manuscript.

## References

[1] ZERBAXA (ceftolozane‐tazobactam) [package insert]. Whitehouse Station, NJ: Merck & Co., Inc.; 2015.

[2] Cluck D , Lewis P , Stayer B , Spivey J , Moorman J . Ceftolozane‐tazobactam: a new‐generation cephalosporin. Am J Health Syst Pharm. 2015;72:2135‐2146. 26637512 10.2146/ajhp150049

[3] Farrell DJ , Sader HS , Flamm RK , Jones RN . Ceftolozane/tazobactam activity tested against gram‐negative bacterial isolates from hospitalized patients with pneumonia in US and European medical centres (2012). Int J Antimicrob Agents. 2014;43:533‐539. 24856078 10.1016/j.ijantimicag.2014.01.032

[4] Chandorkar G , Huntington JA , Gotfried MH , et al. Intrapulmonary penetration of ceftolozane/tazobactam and piperacillin/tazobactam in healthy adult subjects. J Antimicrob Chemother. 2012;67:2463‐2469. 22773741 10.1093/jac/dks246

[5] ClinicalTrials.gov . Study of intravenous ceftolozane/tazobactam compared to piperacillin/tazobactam in ventilator‐associated pneumonia. https://clinicaltrials.gov/ct2/show/NCT01853982. Accessed August 27, 2017.

[6] ClinicalTrials.gov . Safety and efficacy study of ceftolozane/tazobactam to treat ventilated nosocomial pneumonia (ASPECT‐NP). https://clinicaltrials.gov/ct2/show/ NCT02070757. Accessed August 27, 2017.

[7] Gelfand MS , Cleveland KO . Ceftolozane/tazobactam therapy of respiratory infections due to multidrug‐resistant *Pseudomonas aeruginosa* . Clin Infect Dis. 2015;61:853‐855. 26021991 10.1093/cid/civ411

[8] Castón JJ , De la Torre Á , Ruiz‐Camps I , et al. Salvage therapy with ceftolozane‐tazobactam for multidrug‐resistant *Pseudomonas aeruginosa* infections. Antimicrob Agents Chemother. 2017;61:e02136‐16. 27956431 10.1128/AAC.02136-16 PMC5328537

[9] Munita JM , Aitken SL , Miller WR , et al. Multicenter evaluation of ceftolozane/tazobactam for serious infections caused by carbapenem‐resistant *Pseudomonas aeruginosa* . Clin Infect Dis. 2017;65:158‐161. 28329350 10.1093/cid/cix014 PMC5850333

[10] Soliman R , Lynch S , Meader E , Pike R , Turton JF , Hill RLR , Woodford N , Livermore DM , et al. Successful ceftolozane/tazobactam treatment of chronic pulmonary infection with pan‐resistant *Pseudomonas aeruginosa* . JMM Case Rep. 2015;2: https://doi.org/10.1099/jmmcr.0.000025.

[11] Fraile‐Ribot PA , Mulet X , Cabot G , et al. In vivo emergence of resistance to novel cephalosporin‐β‐lactamase inhibitor combinations through the duplication of the amino acid D149 from OXA‐2 β‐lactamase (OXA‐539) in ST235 *Pseudomonas aeruginosa* . Antimicrob Agents Chemother. 2017;61:e01117‐17. 28674059 10.1128/AAC.01117-17 PMC5571340

[12] Oliver A , Mulet X , López‐Causapé C , Juan C . The increasing threat of *Pseudomonas aeruginosa* high‐risk clones. Drug Resist Updat. 2015;21–22:41‐59. 10.1016/j.drup.2015.08.002 26304792

[13] Haidar G , Philips NJ , Shields RK , et al. Ceftolozane‐tazobactam for the treatment of multidrug‐resistant *Pseudomonas aeruginosa* infections: clinical effectiveness and evolution of resistance. Clin Infect Dis. 2017;65:110‐120. 29017262 10.1093/cid/cix182 PMC5848332

[14] Mandell LA , Wunderlink RG , Anzueto A , et al. Infectious diseases Society of America/American Thoracic Society consensus guidelines on the management of community‐acquired pneumonia in adults. Clin Infect Dis. 2007;44:S27‐S72. 17278083 10.1086/511159 PMC7107997

[15] Pierson DJ , Horton CA , Bates PW . Persistent bronchopleural air leak during mechanical ventilation. A review of 39 cases. Chest. 1986;90:321‐323. 3743142 10.1378/chest.90.3.321

[16] Kazmierczak KM , Rabine S , Hackel M , et al. Multiyear, multinational survey of the incidence and global distribution of metallo‐β‐lactamase‐producing enterobacteriaceae and *Pseudomonas aeruginosa* . Antimicrob Agents Chemother. 2015;60:1067‐1078. 26643349 10.1128/AAC.02379-15 PMC4750703

[17] Alatoom A , Elsayed H , Lawlor K , et al. Comparison of antimicrobial activity between ceftolozane‐tazobactam and ceftazidime‐avibactam against multidrug‐resistant isolates of *Escherichia coli*,* Klebsiella pneumoniae*, and *Pseudomonas aeruginosa* . Int J Infect Dis. 2017;62:39‐43. 28610832 10.1016/j.ijid.2017.06.007

[18] Berrazeg M , Jeannot K , Ntsogo Enguéné VY , et al. Mutations in β‐lactamase AmpC increase resistance of *Pseudomonas aeruginosa* isolates to antipseudomonal cephalosporins. Antimicrob Agents Chemother. 2015;59:6248‐6255. 26248364 10.1128/AAC.00825-15 PMC4576058

[19] Rodríguez‐Martínez JM , Poirel L , Nordmann P . Molecular epidemiology and mechanisms of carbapenem resistance in *Pseudomonas aeruginosa* . Antimicrob Agents Chemother. 2009;53:4783‐4788. 19738025 10.1128/AAC.00574-09 PMC2772299

